# Preparation of Microparticles Capable of Glucose-Induced Insulin Release under Physiological Conditions

**DOI:** 10.3390/polym10101164

**Published:** 2018-10-18

**Authors:** Kentaro Yoshida, Kazuma Awaji, Seira Shimizu, Miku Iwasaki, Yuki Oide, Megumi Ito, Takenori Dairaku, Tetsuya Ono, Yoshitomo Kashiwagi, Katsuhiko Sato

**Affiliations:** 1 School of Pharmaceutical Sciences, Ohu University 31-1 Misumido, Tomita-Machi, Koriyama, Fukushima 963-8611, Japan; t-dairaku@pha.ohu-u.ac.jp (T.D.); t-ono@pha.ohu-u.ac.jp (T.O.); y-kashiwagi@pha.ohu-u.ac.jp (Y.K.); 2 Graduate School of Pharmaceutical Sciences, Tohoku University, 6-3 Aoba, Aramaki, Aoba-ku, Sendai 980-8578, Japan; sabapena3@yahoo.co.jp (K.A.); simizuseira@gmail.com (S.S.); ilax84@gmail.com (M.I.); cyluaurkiinet@gmail.com (Y.O.); migumeito@gmail.com (M.I.); satok@m.tohoku.ac.jp (K.S.)

**Keywords:** phenyl boronic acid, hydrogen peroxide, drug delivery system, insulin, LbL film, glucose response

## Abstract

Hydrogen peroxide (H_2_O_2_)-sensitive layer-by-layer films were prepared based on combining phenyl boronic acid (PBA)-modified poly(allylamine) (PAH) with shikimic acid (SA)-modified-PAH through boronate ester bonds. These PBA-PAH/SA-PAH multilayer films could be prepared in aqueous solutions at pH 7.4 and 9.0 in the presence of NaCl. It is believed that the electrostatic repulsion between the SA-PAH and PBA-PAH was diminished and the formation of ester bonds between the SA and PBA was promoted in the presence of NaCl. These films readily decomposed in the presence of H_2_O_2_ because the boronate ester bonds were cleaved by an oxidation reaction. In addition, SA-PAH/PBA-PAH multilayer films combined with glucose oxidase (GOx) were decomposed in the presence of glucose because GOx catalyzes the oxidation of D-glucose to generate H_2_O_2_. The surfaces of CaCO_3_ microparticles were coated with PAH/GOx/(SA-PAH/PBA-PAH)_5_ films that absorbed insulin. A 1 mg quantity of these particles released up to 10 μg insulin in the presence 10 mM glucose under physiological conditions.

## 1. Introduction

Stimuli-sensitive drug release devices such as microchips [1], gels [2,3] and microcapsules [4,5] have been widely studied with regard to biomedical applications. Glucose-sensitive insulin release has attracted particular attention, since the development of insulin delivery systems for the self-regulation of blood glucose levels would be helpful for diabetes mellitus patients. Glucose-sensitive materials such as lectins [6], glucose oxidase [7,8] and phenyl boronic acid (PBA) [9,10,11] have been employed for this purpose. Because the glucose-sensitive release of insulin could mimic the function of the pancreas, this technology might eliminate repetitive insulin injections.

Layer-by-layer (LbL) multilayer films can be prepared by the alternating and repeated deposition of polymeric materials on the surface of a solid substrate [12]. The polymers are deposited on the substrate by attractive forces that include electrostatic interaction [13,14,15], hydrogen bonds [16,17], avidin-biotin binding [18] and sugar-lectin binding [19]. Thus, both synthetic polymers and biological materials can be used to produce LbL films, with applications in sensors [20,21], separation and purification [22,23], microcapsules [24,25,26], drug delivery systems (DDSs) [27,28,29] and stimuli-sensitive devices [30,31]. There have been many studies devoted to stimuli-sensitive LbL films that decompose in response to environmental stimuli such as pH [32], ionic strength [33], biological molecules [34] and electrical signals [35]. If such films can incorporate glucose-sensitive materials, a release process based on physiological conditions could be realized. Therefore, it has been suggested that glucose-sensitive multilayer films could be used to develop insulin delivery systems.

**Figure 1:**
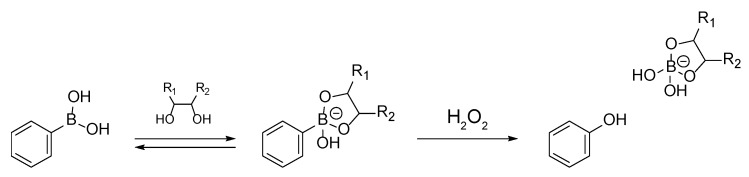
Sequential equilibrium and oxidation reactions of phenyl boronic acid with a diol and H_2_O_2_, respectively.

Our own groups have previously developed pH- and sugar-sensitive LbL films composed of PBA-modified poly(allylamine hydrochloride) (PBA-PAH) and poly(vinyl alcohol) (PVA) [36]. PBA derivatives are of particular interest because they selectively bind 1,2- and 1,3-diol compounds such as sugars [37,38]; Figure 1 shows the binding of a diol compound to a boronate ester. For this reason, the use of PBA derivatives in the development of sugar-sensitive drug delivery systems has been suggested [39,40,41,42]. It is difficult to decompose these PBA-PAH/PVA LbL films under physiological conditions, but PBA and boronate esters are sensitive to reactive oxygen species (ROS). As an example, these compounds can be irreversibly decomposed as the result of the oxidative scission of the carbon-boron bond by hydrogen peroxide (H_2_O_2_) [43]. We have previously reported the H_2_O_2_-induced decomposition of LbL films consisting of PBA-PAH and PVA [44], as well as the glucose-induced decomposition of multilayer PBA-PAH/PVA films by glucose oxidase (GOx) [45]. 

**Figure 2:**
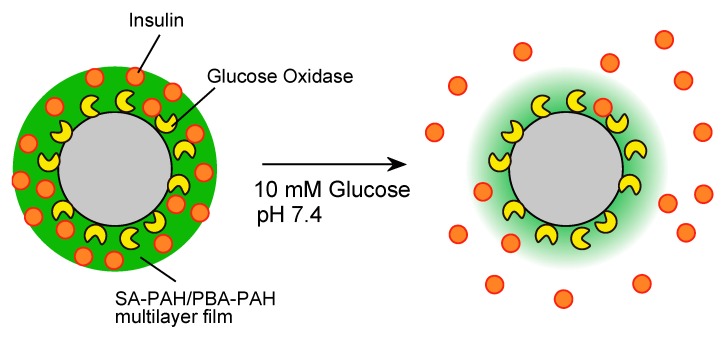
A schematic illustration of the functioning of a glucose-induced insulin release microparticle.

Microparticles coated with LbL films are extremely useful in DDS applications, although such materials made with PBA-PAH/PVA multilayer films have a tendency to aggregate. In the present work, LbL films capable of undergoing H_2_O_2_- and glucose-induced decomposition were applied as coatings to fine particles, with the aim of developing a system for glucose-induced insulin release under physiological conditions (Figure 2)

## 2. Experimental

### 2.1. Materials

PAH (molecular weight: 150,000) and insulin (human, recombinant) were obtained from Nittobo Co. (Tokyo, Japan) and Wako Pure Chemical Ind. (Osaka, Japan), respectively. GOx (activity: 100 units mg^−1^ solid) was obtained from Toyobo Co., Ltd. (Osaka, Japan) and H_2_O_2_ (30% aqueous solution) was obtained from Santoku Chemical Industries Co., Ltd. (Tokyo, Japan). Shikimic acid (SA), 4-carboxyphenyl boronic acid and 4-(*N*,*N*-dimethylaminosulfonyl)-7-(2-aminoethylamino)-2,1,3-benzoxadiazole (DBD-ED) were purchased from Tokyo Chemical Industry Co., Ltd. (Tokyo, Japan) while 1-ethyl-3-(3-dimethylaminopropyl)carbodiimide hydrochloride (EDC) and N-hydroxysuccinimide (NHS) were obtained from Nacalai Tesque Co. (Kyoto, Japan). All other reagents were of the highest possible grade and were used as received without further purification.

PBA-PAH was synthesized according to a literature procedure [36]. The PAH contained approximately 15% PBA residues (based on the molar ratio of PBA to primary amine groups) as calculated from the proportions of nitrogen and carbon determined by elemental analysis. The calculated elemental composition of the PBA-PAH was C = 39.99%, H = 7.55% and N = 11.53%, and the experimental results showed C = 42.29%, H = 6.2% and N = 11.42%. 

SA-modified PAH (SA-PAH) was synthesized by reacting SA and PAH in water in the presence of NHS and EDC. In this process, EDC (109 mg) was added to a solution of SA (100 mg), PAH (100 mg) and NHS (66 mg) in water (30 mL) and the reaction mixture was stirred for 1 h at 0 °C and then for 12 h at room temperature. The resulting SA-PAH was purified by dialysis in water using a dialysis membrane (molecular weight cut-off of 12,000–14,000, Fast Gene, Nippon Genetics). The PAH contained approximately 26% SA residues (based on the molar ratio of SA to primary amine groups) as calculated from the proportions of nitrogen and carbon determined by elemental analysis. The calculated elemental composition of the SA-PAH was C = 47.00%, H = 7.81% and N = 11.32%. The C /N ratio based on these data is 4.15, and so SA-PAH bearing 26% SA was obtained. 

**Figure 3:**
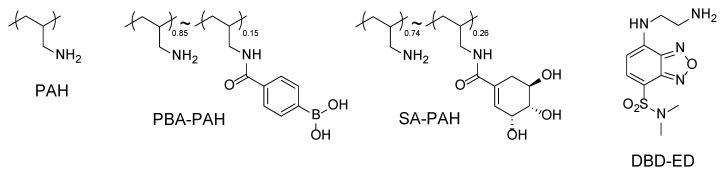
The chemical structures of the PAH, PBA-PAH, SA-PAH and DBD-ED.

DBD-labeled insulin (DBD-insulin) was synthesized by reacting DBD-ED and insulin in water in the presence of NHS and EDC. EDC (3.30 mg) was added to a solution of DBD-ED (4.91 mg) in a mixture of DMF (0.5 mL), insulin (100 mg) and NHS (1.98 mg) in water. The reaction mixture was stirred for 1 h at 0 °C and then for 12 h at room temperature. The resulting DBD-insulin was purified by dialysis in water using a dialysis membrane, after which it was freeze-dried. The chemical structures of the PBA-PAH, SA-PAH and DBD-ED are shown in Figure 3. 

### 2.2. Apparatus

A quartz crystal microbalance (QCM, QCA 917 system, Seiko EG & G, Tokyo, Japan) and a flow cell were used for gravimetric analysis of the LbL films. A 9 MHz AT-cut quartz resonator coated with a thin Au layer (surface area: 0.2 cm^2^) was used as a probe, such that the adsorption of 1 ng of a substance induced a −0.91 Hz change in the resonance frequency. UV-vis and fluorescence spectra were acquired using a 3100PC spectrophotometer (Shimadzu, Kyoto, Japan) and an FP-6500 fluorescence spectrophotometer (JASCO, Tokyo, Japan), respectively. Optical microscopy and fluorescence microscopy images were obtained with an LSM510 instrument (ZEISS, Oberkochen, Germany).

### 2.3. Preparation of SA-PAH/PBA-PAH Multilayer Films

SA-PAH/PBA-PAH multilayer films were prepared on solid substrates. Each substrate was initially immersed in an SA-PAH solution (0.1 mg/mL) for 15 min to deposit the first SA-PAH layer. After rinsing in a working buffer for 5 min to remove any weakly adsorbed SA-PAH, the substrate was immersed in a PBA-PHA solution (0.1 mg/mL) for 15 min to deposit PBA-PHA. The second SA-PAH and PBA-PHA layers were deposited using the same technique and this deposition process was repeated to build an (SA-PAH/PBA-PAH)_10_ multilayer film. The working buffers consisted of 10 mM 2-(*N*-morpholino)ethanesulfonic acid (MES) buffer (pH 5.0), 10 mM 4-(2-hydroxyethyl)-1-piperazineethanesulfonic acid (HEPES) buffer (pH 7.4) or 10 mM *N*-Cyclohexyl-2-aminoethanesulfonic acid (CHES) buffer (pH 9.0). The effect of ionic strength on the SA-PAH/PBA-PAH multilayer films was investigated by adding NaCl to the buffers.

SA-PAH/PBA-PAH films containing GOx was prepared by building (SA-PAH/PBA-PAH)_10_ multilayer films on PAH/GOx films at ambient temperature (approximately 20 °C). A solid substrate was coated in advance with a PAH/GOx film by immersing the slide alternately in 0.1 mg mL^−1^ PAH and 0.1 mg mL^−1^ GOx solutions [39]. The glucose response was studied in a series of 10 mM HEPES buffers (pH 7.4, 150 mM or 1 M NaCl) and 10 mM CHES buffer (pH 9.0, 1 M NaCl). A 9 MHz Au-coated quartz resonator and a quartz slide (4.5 × 0.9 × 0.1 cm) were used for QCM analysis and UV-vis absorption measurements, respectively. 

### 2.4. H_2_O_2_- and Glucose-Induced Decomposition of LbL Films 

The H_2_O_2_- and glucose-induced decompositions of various films were studied by UV-vis absorption spectroscopy. To determine the kinetics of film decomposition, one side of a quartz slide was coated with an LbL film and the slide was placed in a quartz cuvette (optical path length: 10 mm) filled with a buffer solution. The slide was placed near the sidewall of the cuvette, parallel to the light path, in order to avoid blocking the incident light. The absorbance of the solution at 255 nm was monitored with gentle stirring of the buffer to estimate the extent of film decomposition at different pH values in the absence and presence of H_2_O_2_ and glucose.

### 2.5. Preparation of Microparticles Coated with SA-PAH/PBA-PAH Multilayer Films

In this procedure, 10 mL of a Ca(NO_3_)_2_ (472 mg) solution was combined with 10 mL of a Na_2_CO_3_ (210 mg) solution and the mixture was stirred for 30 min, after which the precipitated CaCO_3_ particles were collected by centrifugation and washed with water [46]. The surfaces of these particles was coated with multilayer films by immersing them alternately in 0.1 mg/mL SA-PAH and 0.1 mg/mL PBA-PAH solutions (both in a pH 9.0 10 mM CHES buffer) for 15 min. After each deposition, the CaCO_3_ particles were collected by centrifugation and rinsed in the working buffer for 5 min. The alternating deposition of SA-PAH and PBA-PAH was repeated in this manner to prepare (SA-PAH/PBA-PAH)_5_ multilayer films on the CaCO_3_ particles. 

Glucose-sensitive microparticles coated with SA-PAH/PBA-PAH multilayer films were prepared by building (SA-PAH/PBA-PAH)_5_ multilayer films on PAH/GOx film-coated CaCO_3_ particles. The CaCO_3_ particles were initially coated with a PAH/GOx film by immersing them alternately in 0.1 mg mL^−1^ PAH and 0.1 mg mL^−1^ GOx solutions. Microparticles containing DBD-insulin were prepared using a PBA-PAH solution with 0.1 mg/mL DBD-insulin added. These glucose-sensitive films were prepared in a 10 mM HEPES buffer (pH 7.4, 150 mM NaCl) and were freeze-dried after being prepared.

### 2.6. H_2_O_2_- and Glucose-Induced Decomposition of Microparticles Coated with LbL Films

H_2_O_2_-sensitive microparticles coated with SA-PAH/PBA-PAH multilayer films were assessed using UV-vis absorption spectroscopy (UV-3100PC, Shimazu Co., Kyoto, Japan). The freeze dried microparticles (20 mg) were immersed in solutions containing from 0.1 to 10 mM H_2_O_2_ and stirred. After a specific time period, the microparticles were removed by centrifugation and the supernatant was collected. The H_2_O_2_ in the supernatant was decomposed by adding 1.0 mg/mL catalase (100 μL) and the absorbance of the supernatant at 255 nm was monitored to determine the amount of PBA in solution due to decomposition of the SA-PAH/PBA-PAH multilayer films.

The DBD-insulin release from glucose-sensitive PAH/GOx/(SA-PAH/PBA-PAH)_5_ film-coated-microparticles were studied by fluorescence emission spectroscopy. The freeze-dried microparticles (10 mg) were immersed in solutions containing 1 to 100 mM glucose and stirred for 60 min. The microparticles were subsequently removed by centrifugation and the supernatant was collected. The supernatant was passed through a filter (pore size 0.45 μm, RC-membrane, Sartorius Stedim, Germany) and the fluorescence intensity of the filtrate was recorded at 505 nm (excitation: 488 nm) to estimate the amount of DBD-insulin released.

## 3. Results and Discussion

### 3.1. SA-PAH/PBA-PAH Multilayer Films Characterization

**Figure 4:**
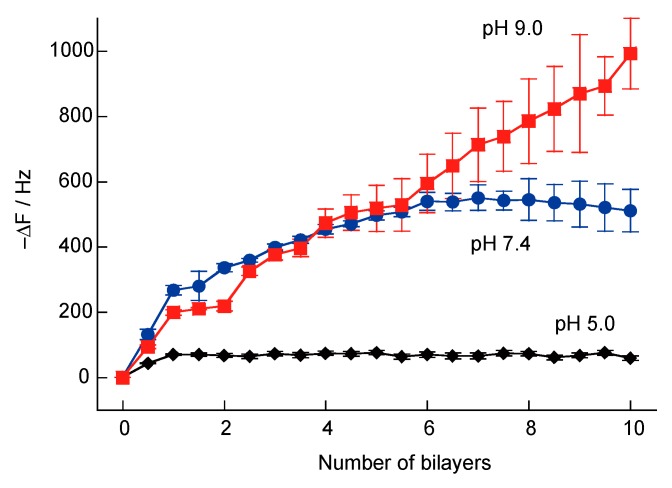
QCM resonator frequency changes during the preparation of (SA-PAH/PBA-PAH)_n_ films in a 10 mM CHES buffer (pH 9.0) in the presence and absence of NaCl (150 mM or 1 M).

In a previous study, we attempted to prepare H_2_O_2_- and glucose-responsive multilayer films composed of microparticles coated with PBA-PAH and PVA [36]. However, microparticles coated with PBA-PAH/PVA multilayer films were found to aggregate. Multilayer films composed of PBA-PAH were found to accumulate positive charges, while the PVA films had low charge densities. Therefore, aggregation of the particles was attributed to a reduction in the repulsive force between them. In the present study, H_2_O_2_-responsive multilayer films composed of SA-PAH and PBA-PAH were prepared via the formation of boronate ester bonds between the boronic acid moieties in the PBA-PAH and the diol units in the SA. However, the deposition of SA-PAH/PBA-PAH multilayer films could potentially be hindered by the electrostatic repulsion between the SA-PAH and PBA-PAH. For this reason, increasing the ionic strength of the associated solutions was assessed as a means of promoting film formation. The effect of ionic strength (as reflected by changes in the NaCl concentration) during the preparation of SA-PAH/PBA-PAH multilayer films was investigated using QCM (Figure 4). Variations in the resonance frequency (ΔF) were monitored after rinsing with the working buffer, and −ΔF was found to increase when the quartz resonator was exposed to the SA-PAH and PBA-PAH solutions in the presence of NaCl, indicating that SA-PAH/PBA-PAH multilayer films were successfully formed on the surface of the quartz resonator. In contrast, SA-PAH/PBA-PAH multilayers could not be prepared in the absence of NaCl. The deposition densities of the (SA-PAH/PBA-PAH)_10_ film were calculated to be 2.80 and 5.45 µg cm^−2^ when using the 150 mM and 1 M NaCl solutions, respectively. Thus, as expected, higher NaCl concentrations increased the amount of SA-PAH/PBA-PAH multilayer film that was deposited. This likely occurred because the electrostatic repulsion between the SA-PAH and PBA-PAH was diminished, promoting the formation of ester bonds between the shikimic and boronic acid moieties. A high ionic strength in the buffer also causes the polyelectrolyte to contract so as to increase the amount of polymer deposited per layer [47]. 

**Figure 5:**
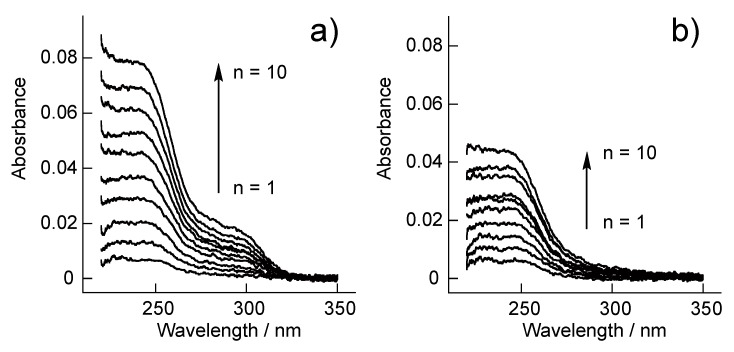
UV-vis absorption spectra during the preparation of (SA-PAH/PBA-PAH)_n_ films in a 10 mM CHES buffer (pH 9.0) in the presence of 1 M NaCl (**a**) or 150 mM NaCl (**b**).

Figure 5 shows the UV-vis absorption spectra of SA-PAH/PBA-PAH multilayer films prepared on quartz slides. These spectra exhibit adsorption maxima at approximately 242 nm, originating from the PBA units in the PBA-PAH. The intensity of these peaks increased with increasing number of depositions, suggesting that SA-PAH/PBA-PAH multilayer films were successfully prepared on the slides. In agreement with the QCM results, increasing the NaCl concentration of the solution increased the amount of PBA-PAH deposited.

**Figure 6:**
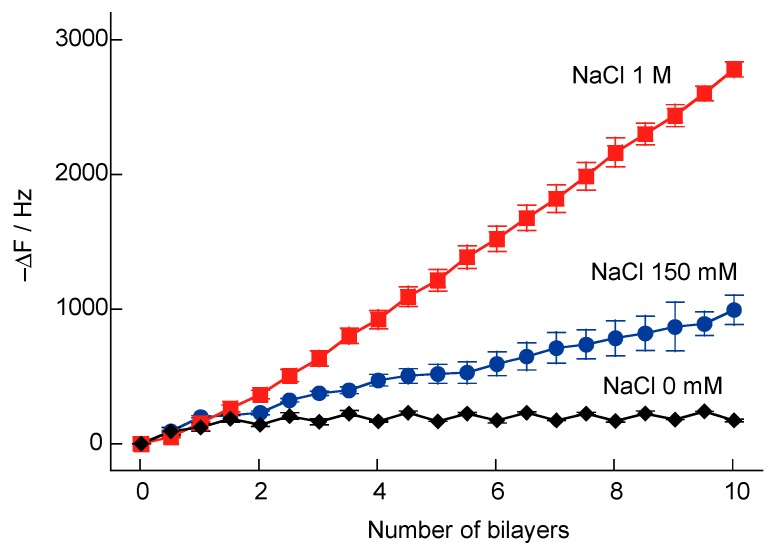
The effect of the pH value during the preparation of SA-PAH/PBA-PAH multilayer films in a 10 mM MES (pH 5.0), 10 mM HEPES (pH 7.4) or 10 mM CHES buffer (pH 9.0). All buffer solutions contained 150 mM NaCl.

The bonding of PBA to diols is known to be unstable at neutral and acidic pH values [31,32]. Therefore, we evaluated the stability of the SA-PAH/PBA-PAH multilayer films in aqueous solutions with different pH values. Figure 6 presents the −ΔF values determined for SA-PAH/PBA-PAH multilayer films at various pH values. Films were successfully prepared at pH values of 7.4 and 9.0 but not at pH 5. Under low pH conditions, bonding between the PBA and diols decreased and the formation of multilayer film was made more difficult [37,38] as a result of the electrostatic repulsion between the SA-PAH and PBA-PAH.

**Figure 7:**
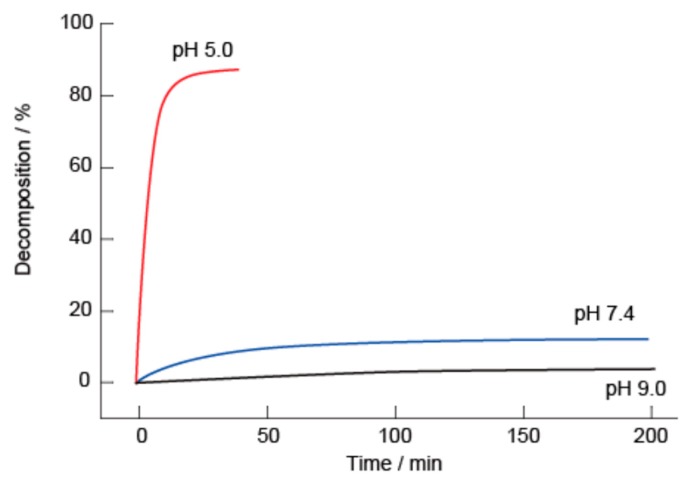
Decomposition kinetics of (SA-PAH/PBA-PAH)_10_ films in 10 mM MES (pH 5.0), 10 mM HEPES (pH 7.4) or 10 mM CHES (pH 9.0) buffers. The films were prepared using a 10 mM CHES buffer (pH 9.0). All buffer solutions contained 1 M NaCl.

Figure 7 summarizes the kinetics of the decomposition of the (SA-PAH/PBA-PAH)_10_ films in solutions with pH values of 5.0, 7.4 and 9.0. The percentage decomposition was estimated from the changes in the absorption intensity at 255 nm. The films evidently decomposed rapidly at pH 5.0, presumably because the bonding between the PBA and diols decomposed under weakly acidic conditions. In contrast, the films were stable at pH 9.0 for 3 h, and only 10% decomposition was observed at pH 7.4 over 3 h. It should be noted that the evident stability at pH 7.4 suggests that such films have potential applications in biomedical devices. Similar pH-dependent stability has been reported for PVA/PBA-poly(amidoamine) dendrimer films [48]. 

**Figure 8:**
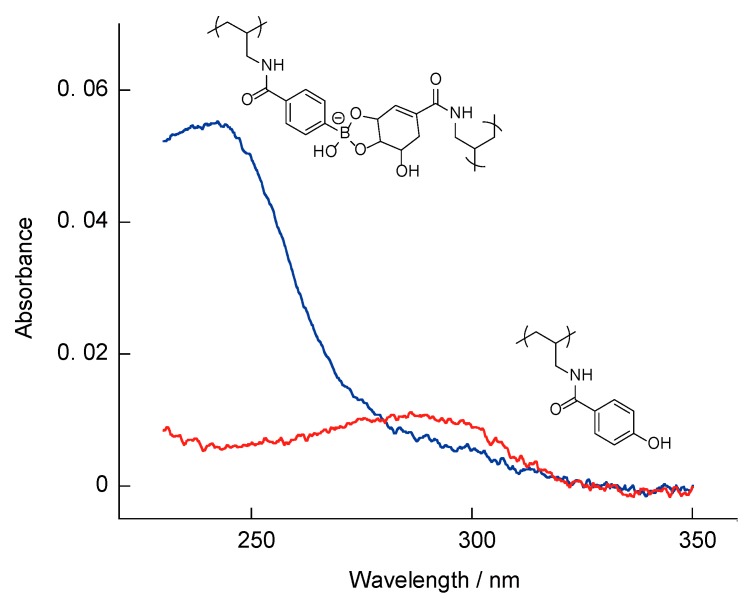
UV-vis absorption spectra of a (SA-PAH/PBA-PAH)_10_ film before (Blue line) and after 60 min immersion in 1 mM H_2_O_2_ (red line). The film was prepared in a 10 mM CHES buffer (pH 9.0) in the presence of 1 M NaCl.

Figure 8 presents the UV-vis absorption spectra of (SA-PAH/PBA-PAH)_10_ films before and after 60 min immersion in 1 mM H_2_O_2_. Due to PBA, the absorption intensity at 242 nm decreased markedly over time during this process. As shown in Figure 1, the carbon-boron bonds in PBA and boronate esters are oxidatively cleaved by H_2_O_2_, suggesting that the H_2_O_2_ induced the decomposition of the (SA-PAH/PBA-PAH)_10_ films. The absorption at 290 nm also increases slightly over time in these spectra, due to the formation of phenol resulting from the oxidation of PBA.

**Figure 9:**
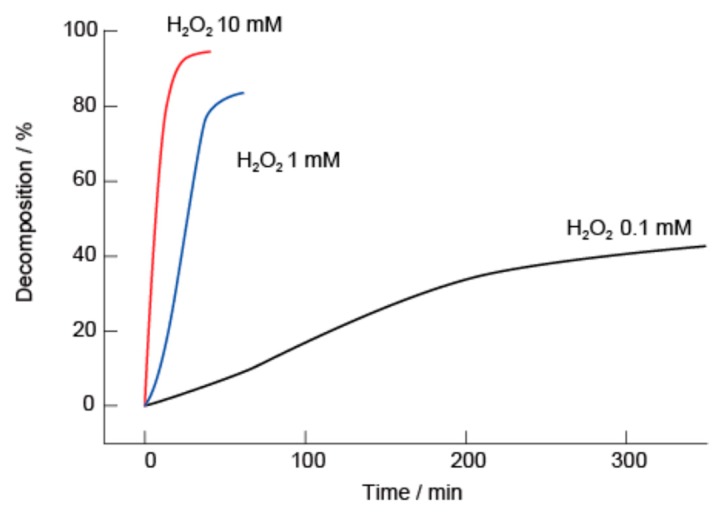
Decomposition kinetics of (SA-PAH/PBA-PAH)_10_ films in the presence of 0.1, 1 and 10 mM H_2_O_2_ in a 10 mM CHES buffer (pH 9.0). The films were prepared in a 10 mM CHES buffer (pH 9.0). All buffer solutions contained 1 M NaCl.

The decomposition kinetics of the (SA-PAH/PBA-PAH)_10_ films in the presence of H_2_O_2_ was assessed by monitoring the adsorption intensity at 255 nm (Figure 9). This wavelength represents an isosbestic point for a combination of PBA-PAH and PBA-PAH during oxidation by H_2_O_2_. The (SA-PAH/PBA-PAH)_10_ film was found to decompose markedly in the presence of H_2_O_2_ and the rate of decomposition increased with increases in the H_2_O_2_ concentration. As an example, the decomposition percentages of films were determined to be 13%, 81% and 94% after 60, 60 and 20 min in the presence of 0.1, 1 and 10 mM H_2_O_2_, respectively.

**Figure A1:**
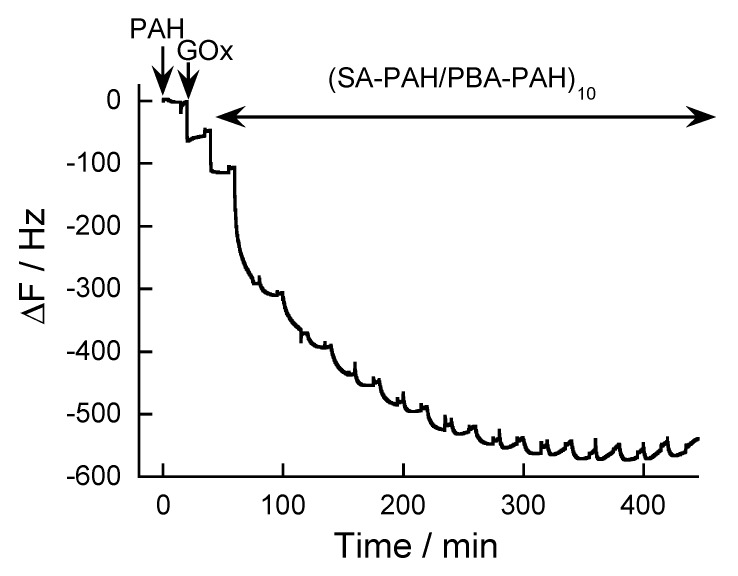
QCM resonator frequency changes during the preparation of a (PAH/GOx) (SA-PAH/PBA-PAH)_10_ film in a 10 mM HEPES buffer (pH 7.4) in the presence of 150 mM NaCl.

A (SA-PAH/PBA-PAH)_10_ film was prepared on a quartz resonator modified with GOx to develop a glucose-responsive unit. GOx was adsorbed into the PAH layer and PBA-PAH layer via electrostatic interactions due to the negative charges present on the GOx at neutral pH (as a result of an isoelectric point of pH 4.2) [49]. The QCM data confirmed that an LbL film could be prepared on a GOx film (see Appendix A Figure A1). Table 1 summarizes the data related to the decomposition of PAH/GOx/(SA-PAH/PBA-PAH)_10_ films after immersion for 60 min in glucose solutions, based on ΔF values obtained by QCM. These data demonstrate that the decomposition of the multilayer films was affected by the glucose concentration. The PAH/GOx/(SA-PAH/PBA-PAH)_10_ films were decomposed because GOx catalyzed the oxidation of D-glucose to generate H_2_O_2_ (Equation (1)).

D-glucose + O_2_ → D-glucono-δ-lactose + H_2_O_2_ (1)

In contrast, two negative decomposition values were obtained in the presence of 1 M NaCl and the values were all negative when using pH 9.0 and 1 mM NaCl. These values are attributed to decreases in the GOx activity due to changes in the ionic strength and basicity of the solutions. Also, glucose was evidently adsorbed on the PBA in the films, thus actually increasing the film masses.

### 3.2. Preparation of Microparticles Coated with SA-PAH/PBA-PAH Multilayer Films

**Figure 10:**
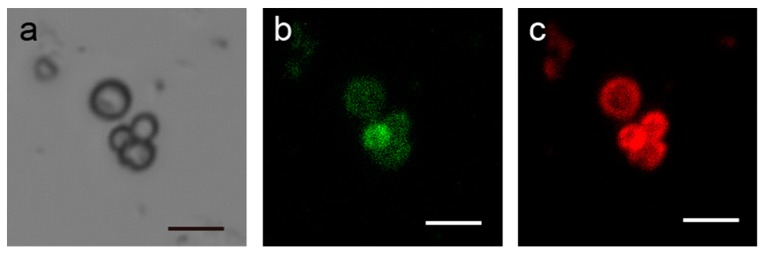
Optical microscopy (**a**) and fluorescence microscopy ((**b**), excitation: 488 nm) and ((**c**) excitation: 543 nm) images of PAH/GOx/(SA-PAH/PBA-PAH)_5_ film-coated CaCO_3_ microparticles in which PBA-PAH and GOx have been labeled with FITC and TRITC, respectively. The scale bar is 5 μm.

Microparticles coated with LbL films were obtained via the deposition of polymers on CaCO_3_ microparticles [46]. Figure 10 shows optical and fluorescence microscopy images of PAH/GOx/(SA-PAH/PBA-PAH)_5_ film-coated CaCO_3_ microparticles in which the PBA-PAH and GOx units have been labeled with fluorescein isothiocyanate (FITC) and tetramethylrhodamine isothiocyanate (TRITC). It is evident that there was no aggregation of the particles during the film deposition process, and that it was possible to apply multilayer films to the particles. The fluorescence images confirm that both PBA-PAH and GOx were deposited on the CaCO_3_.

**Figure 11:**
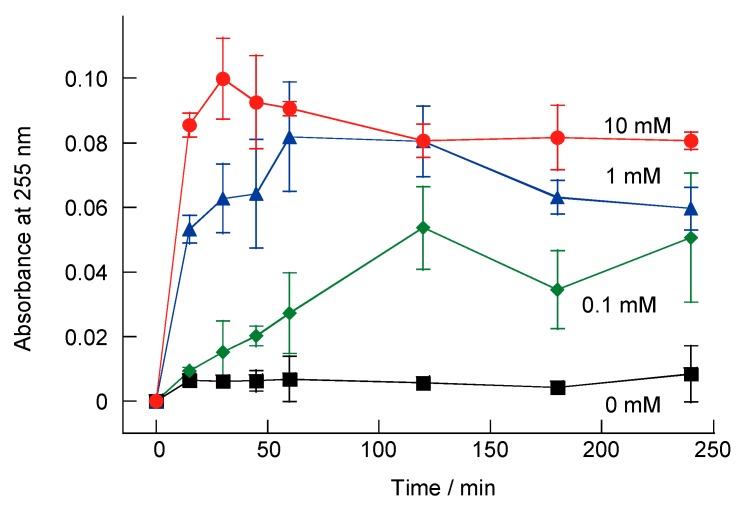
Absorbance at 255 nm resulting from the H_2_O_2_-induced decomposition of (SA-PAH/PBA-PAH)_5_ films on CaCO_3_ microparticles in a 10 mM CHES buffer (pH 9.0) containing 1 M NaCl in the presence of 0, 0.1, 1 or 10 mM H_2_O_2_.

Figure 11 plots the absorbance at 255 nm following the H_2_O_2_-induced decomposition of (SA-PAH/PBA-PAH)_5_ film-coated CaCO_3_ microparticles. This wavelength represents the isosbestic point of PBA-PAH and PBA-PAH oxidized by H_2_O_2_. The absorbance values increased significantly in the presence of H_2_O_2_, with absorbance values of 0.027, 0.082 and 0.091 in conjunction with exposure to H_2_O_2_ concentrations of 0.1, 1 and 10 mM for 1 h. The increase in absorbance is derived from PBA-PAH oxidized by H_2_O_2_, which is a decomposition component of the (SA-PAH/PBA-PAH)_5_ film on the CaCO_3_ microparticles. On the other hand, the multilayer films were not decomposed in the absence of H_2_O_2_. It was found that the SA-PAH/PBA-PAH multilayer film on CaCO_3_ microparticles decomposed in the presence of H_2_O_2_.

**Figure 12:**
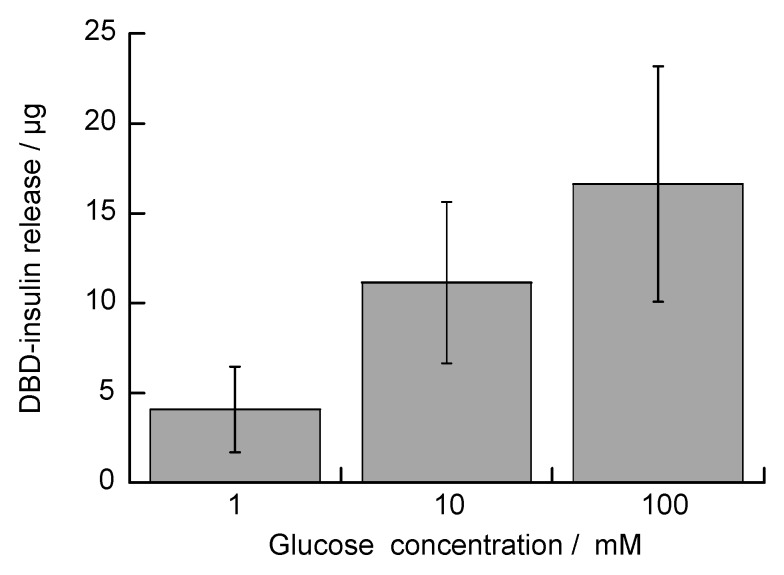
Amounts of DBD-insulin released from 1 mg quantities of PAH/GOx/(SA-PAH/PBA-PAH)_5_ film-coated CaCO_3_ microparticles in a 10 mM HEPES buffer (pH 7.4, containing 150 mM NaCl) in the presence of 1, 10 or 100 mM glucose.

This work also developed microparticles capable of the glucose-induced release of insulin via the catalytic reaction of GOx under physiological conditions. Glucose-sensitive microparticles containing insulin were prepared by adsorbing DBD-insulin on multilayer films. Because insulin has a net negative charge at pH 7.4 (as the isoelectric point of insulin is 5.4 [50]), it will be adsorbed on the PAH layer of the LbL film [51,52]. The glucose-induced decomposition of SA-PAH/PBA-PAH films would therefore be expected to release the insulin adsorbed on the microparticles. Figure 12 shows the DBD-insulin release from 1 mg of PAH/GOx/(SA-PAH/PBA-PAH)_5_ film-coated CaCO_3_ microparticles in glucose solutions of varying concentrations, as calculated from fluorescence intensities of DBD. The amounts of DBD-insulin released were determined to be 0.40, 0.85 and 2.32 μg following the exposure of CaCO_3_ microparticles coated with PAH/GOx/(SA-PAH/PBA-PAH)_5_ films to 1, 10 and 100 mM glucose solutions for 1 h at pH 7.4. DBD-insulin was also found to be released from PAH/GOx/(SA-PAH/PBA-PAH)_5_ film-coated CaCO_3_ microparticles in glucose solution, suggesting that the SA-PAH/PBA-PAH multilayer films on the CaCO_3_ were decomposed because GOx catalyzed the oxidation of D-glucose to generate H_2_O_2_. Once again, the amount of DBD-insulin released from the PAH/GOx/(SA-PAH/PBA-PAH)_5_ film-coated CaCO_3_ microparticles increased with increasing glucose concentration. This process was superior in reactivity to the decomposition of the film under physiological conditions as compared with the method of glucose bound to PBA-PAH/PVA film to cleave the boronate ester bonds [36].

## 4. Conclusions

This work demonstrated that multilayer films can be constructed by the alternating deposition of SA-PBA and PBA-PAH, via the formation of boronate ester bonds. SA-PBA/PBA-PAH films were prepared at pH 7.4 and pH 9 in the presence of NaCl. PAH/GOx/(SA-PBA/PBA-PAH)_n_ films were found to decompose in the presence of glucose due to oxidative scission of the carbon-boron bond of PBA-PAH by enzymatically-generated H_2_O_2_. All steps of the preparation and decomposition of PAH/GOx/(SA-PBA/PBA-PAH)_n_ films could be performed under physiological conditions. In addition, glucose-sensitive microparticles were fabricated by coating CaCO_3_ particles with PAH/GOx/(SA-PBA/PBA-PAH)_5_ films. Insulin pre-absorbed in the PAH/GOx/(SA-PAH/PBA-PAH)_5_ films was released following the addition of glucose, and the extent of release was dependent on the glucose concentration. Again, all steps of this process could be carried out under physiological conditions. These results demonstrate the potential application of microparticles that undergo glucose-induced decomposition in the development of insulin delivery systems. In insulin delivery systems, insulin loading microparticles remain stable at normal levels of blood glucose (~5 mM), and glucose-induced delivery microparticles that release insulin only when the blood glucose is higher than the diabetic level (>10 mM) under physiological conditions are highly desirable [53]. Insulin release system depending on blood glucose will be realized by improving the suitable design of the film composition and the chemical structures of PBA-polymers.

## Figures and Tables

**Table 1:** The decomposition of PAH/GOx/(SA-PAH/PBA-PAH)_10_ films after immersion for 60 min in glucose solutions.

Glucose	pH 7.4	pH 7.4	pH 9.0
	NaCl 150 mM	NaCl 1 M	NaCl 1 M
1 mM	16.9 Hz	−2.9 Hz	−18.9 Hz
10 mM	34.6 Hz	−8.6 Hz	−25.8 Hz
100 mM	43.8 Hz	31.7 Hz	−26.8 Hz
